# Antioxidative Properties of Baltic Sea Keystone Macroalgae (*Fucus vesiculosus*, Phaeophyceae) under Ocean Warming and Acidification in a Seasonally Varying Environment

**DOI:** 10.3390/biology10121330

**Published:** 2021-12-15

**Authors:** Angelika Graiff, Ulf Karsten

**Affiliations:** Institute of Biological Sciences, Applied Ecology and Phycology, University of Rostock, 18059 Rostock, Germany; ulf.karsten@uni-rostock.de

**Keywords:** antioxidative potential, bladder wrack, photosynthetic performance, lipid peroxidation, mesocosm, multifactorial change, seasonal pattern, superoxide dismutase

## Abstract

**Simple Summary:**

The brown seaweed *Fucus vesiculosus* is the dominant and the most ecologically crucial primary producer and habitat founder in the Baltic Sea. In the shallow coastal zone, *F. vesiculosus* is particularly exposed to strongly and rapidly changing environmental conditions due to global change. This study examines how single and joint effects of elevated seawater temperature and pCO_2_ levels influence *F. vesiculosus* in all four seasons, using benthic mesocosms. The antioxidative properties and the sensitivity of *F. vesiculosus* photosynthetic performance to oxidative stress under different global change scenarios were assessed. *F. vesiculosus* tolerated strong hydrogen peroxide stress in all seasons, as reflected in high antioxidative enzyme activities and a low degree of membrane lipid peroxidation. Forecasted warming affected the antioxidative properties of *F. vesiculosus* stronger than acidification, causing significantly increased lipid peroxidation under elevated temperatures in all seasons. However, pCO_2_ levels modulated the oxidative stress of *F. vesiculosus* under warming. Overall, summer heatwaves reaching lethal temperatures in shallow waters will most likely determine the persistence of Baltic *F. vesiculosus*.

**Abstract:**

The keystone macroalga *Fucus vesiculosus* (Phaeophyceae), dominating shallow hard bottom zones, encounters a strongly and rapidly changing environment due to anthropogenic change over the last decades in the Baltic Sea. Thus, in four successive benthic mesocosm experiments, the single and joint effects of increased temperature (Δ + 5 °C) and pCO_2_ (1100 ppm) under ambient irradiances were experimentally tested on the antioxidative properties of western Baltic *F. vesiculosus* in all seasons. The antioxidative properties (superoxide dismutase activity and lipid peroxidation) as well as the sensitivity of *F. vesiculosus* photosynthetic performance (i.e., effective quantum yield) to oxidative stress under these global change scenarios were seasonally examined. *F. vesiculosus* exhibited high and relatively constant photosynthetic performance under artificial hydrogen peroxide (H_2_O_2_) stress in all seasons. High activities of superoxide dismutase and a relatively low degree of the biomarker for lipid peroxidation (malondialdehyde concentration) were found in *F. vesiculosus*. Thus, Baltic *F. vesiculosus* is equipped with a high antioxidative potential to tolerate strong oxidative stress for at least short periods. Antioxidative properties of *F. vesiculosus* were more strongly affected by warming than by acidification, resulting in significantly increased malondialdehyde concentrations under elevated temperature levels in all seasons. Oxidative stress was enhanced in *F. vesiculosus* under warming but seem to be modulated by seasonally varying environmental conditions (e.g., high and low irradiances) and pCO_2_ levels. However, more frequent summer heatwaves reaching and surpassing lethal temperatures in shallow coastal waters may determine the *F. vesiculosus* population’s overall persistence in the Baltic Sea.

## 1. Introduction

Marine seaweeds that inhabit the shallow coastal area of temperate shorelines frequently experience an environment characterized by strong daily and seasonal variations in abiotic drivers, such as high and low irradiances, rapid temperature changes, and pH fluctuations. Due to anthropogenic global change, the continuing warming and its amplification of seasonal fluctuations generates stressful conditions in shallow waters, which seems to be a severe challenge for seaweeds [1,2,3]. The response of seaweeds to unfavorable environmental conditions is mediated through various physiological and biochemical mechanisms, of which the excessive formation and accumulation of reactive oxygen species (ROS) plays a central role by imposing oxidative stress on the cells (reviewed by [4,5,6]).

Perennial seaweeds in temperate latitudes can photosynthesize over broad temperature ranges, as they remain photosynthetically active throughout the year. Various metabolic processes in seaweeds induce ROS formation, particularly photosynthesis. Plants and seaweeds accumulate ROS as a concomitant process of the electron transport systems during photosynthesis and respiration, even under normal metabolic conditions [7]. For instance, the main source of ROS in plant and seaweed tissues is the photosynthetic electron transport system that generates singlet oxygen (^1^O_2_) and superoxide radicals (·O_2_^−^) [8,9]. Additionally, in a sequential reduction of molecular oxygen (O_2_), hydrogen peroxide (H_2_O_2_) as well as hydroxyl radical (·OH) are produced. Various environmental factors, e.g., high or low temperature, rapid temperature changes, nutrient (also carbon) deficiency, high irradiance, and ultraviolet radiation (UVR) stimulate, as a general stress response, the gradual and continued production of ROS. Under stressful environmental conditions, photosynthesis is impaired and surplus energy leads to ROS production [10,11,12,13]. Oxidative stress is recognized as a physiological condition established when ROS formation surpasses the antioxidant defensive systems of organisms, leading to oxidative impairment in lipids, proteins, and DNA [7,14,15]. For instance, a direct indicator of oxidative stress and damage is the level of malondialdehyde (MDA) as a biochemical marker for lipid peroxidation, which is the result of the decomposition of polyunsaturated fatty acids in cell membranes [16]. Thus, MDA often accumulates under oxidative stress, and it has widely been used to distinguish between stressed and unstressed seaweeds (e.g., [13,17,18]).

H_2_O_2_ is omnipresent in the oceans worldwide, with concentrations fluctuating temporally and spatially [19,20,21]. Seawater H_2_O_2_ concentrations between 10 and 300 nM are reported from different water masses and habitats [22,23,24]. In shallow and calm coastal areas, seaweeds may also experience direct oxidative stress where H_2_O_2_ is formed by the photoactivation of dissolved organic material (DOM) due to ultraviolet radiation (UVR) and the release of excited electrons, initiating the reduction of molecular oxygen. The formation of H_2_O_2_ is mainly observed in surface waters or flat water zones characterized by high concentrations of DOM and oxygen [19,25,26,27,28]. In surface waters and/or intertidal pools, H_2_O_2_ can reach even micromolar concentrations (<2 µM; [29,30]). H_2_O_2_ undergoes relatively few reactions with biologically important molecules, but it passes quickly through membranes by diffusion; thus, it is most likely the intracellular preliminary stage for more reactive oxidants (reviewed by Winterbourn [31]). However, H_2_O_2_ directly inhibits photosynthesis by affecting several photosynthetically important enzymes such as RuBisCO [32,33,34].

Seaweeds, however, are equipped with various defense mechanisms that effectively remove ROS. Common antioxidative systems in seaweeds consist of several enzymes, e.g., superoxide dismutase (SOD), ascorbate peroxidase (APX), and catalase (CAT), as well as the antioxidants ascorbic acid, glutathione, β-carotene, and α-tocopherol [11,35,36]. The antioxidative enzymes transform distinct ROS to less toxic compounds. For example, SOD scavenges and produces ROS at the same time because it catalyzes the conversion of ·O_2_^−^ to H_2_O_2_ and oxygen [7]. Then, H_2_O_2_ is further deprotonated by CAT or APX. SOD is known as “the first line of defense” against ROS and plays a central role in the antioxidative system, as it is a powerful scavenger of ·O_2_^−^, which otherwise initiates a ROS cascade [6,37]. Furthermore, seaweeds are also able to detoxify H_2_O_2_ by excreting H_2_O_2_ to reduce intracellular concentrations [38,39]. In recent years, several studies have investigated the underlying molecular processes of ROS acclimation. These include the regulation of gene expression, for example the induction of various ROS scavenging enzymes (e.g., genes encoding for SOD, APX, CAT), heat shock proteins (HSPs), early light-inducible proteins (ELIPs), and the general adjustment of the primary metabolism towards abiotic factors (i.e., high light, high temperature, rapid changes in salinity) in seaweeds [40,41,42,43,44,45]. An overall trend was revealed by Collén et al. [43], who concluded that abiotic stress initiated an increased expression of “stress key genes” (e.g., HSP and ELIP genes), which appear together with decreased expression of energy protein synthesis-related genes. 

Along the rocky and stony shores of the Baltic Sea, the brown seaweed *Fucus vesiculosus* L. forms biomass-rich belts and thereby founds the basis of a productive and structurally complex community [46,47]. *F. vesiculosus* is a keystone species in shallow Baltic coastal habitats, which are characterized by fluctuating environmental conditions, particularly by annual and seasonal variations in pH (7.4–8.5) and temperature (<0 to 20/25 °C), which are tolerated by perennial *F. vesiculosus* [48,49]. The formation and structure of the *F. vesiculosus* ecosystem has been attributed to different abiotic factors and is maintained by fine-tuned biotic interactions [49,50,51,52]. For example, *F. vesiculosus* populations seem to severely suffer from environmental changes over the past decades, as reflected by their decline in abundance and depth penetration along Baltic shores [53,54].

Baltic Sea surface temperature has warmed rapidly during recent decades and is predicted to increase by 3–6 °C until 2100 [55]. Additionally, to this continuous warming trend, short-term extreme warming events known as “marine heat-waves” (*sensu* Hobday et al. [56]) became also more frequent in this region [57]. The simultaneous rise in pCO_2_ and the accompanying acidification of the brackish Baltic Sea is challenging to forecast [58], but model simulations for the Baltic Proper projected a gradually decreasing mean surface pH until the end of this century [59].

Thus, co-occurring changes of environmental variables such as warming and acidification of the Baltic Sea may individually or interactively impact the antioxidative properties of *F. vesiculosus*. Their effect on *F. vesiculosus* may differ seasonally, depending on, for example, growth periods. The present study investigates single and joint effects of increased seawater temperature (Δ + 5 °C) and pCO_2_ (1100 ppm) as predicted for shallow shores until the end of this century in the Baltic Sea [58,60,61] on adult *F. vesiculosus* in all four seasons. To simulate these specific global change scenarios, benthic mesocosms (Kiel Outdoor Benthocosms (KOBs)) were used. Temperature and pCO_2_ elevation were added on top of the natural fluctuations and variabilities of all abiotic factors present in the KOBs [62]. We hypothesized that higher inorganic carbon availability under elevated pCO_2_ may decrease, but higher temperatures may enhance oxidative stress for *F. vesiculosus*. Therefore, the antioxidative properties (SOD activity and lipid peroxidation), as well as the sensitivity of *F. vesiculosus* photosynthetic performance (i.e., effective quantum yield) to artificial oxidative stress resulting from exposure to H_2_O_2_ under these global change scenarios, were examined for the first time.

## 2. Materials and Methods

### 2.1. Experimental Setup and Conditions in the Kiel Outdoor Benthocosms

**Figure 1:**
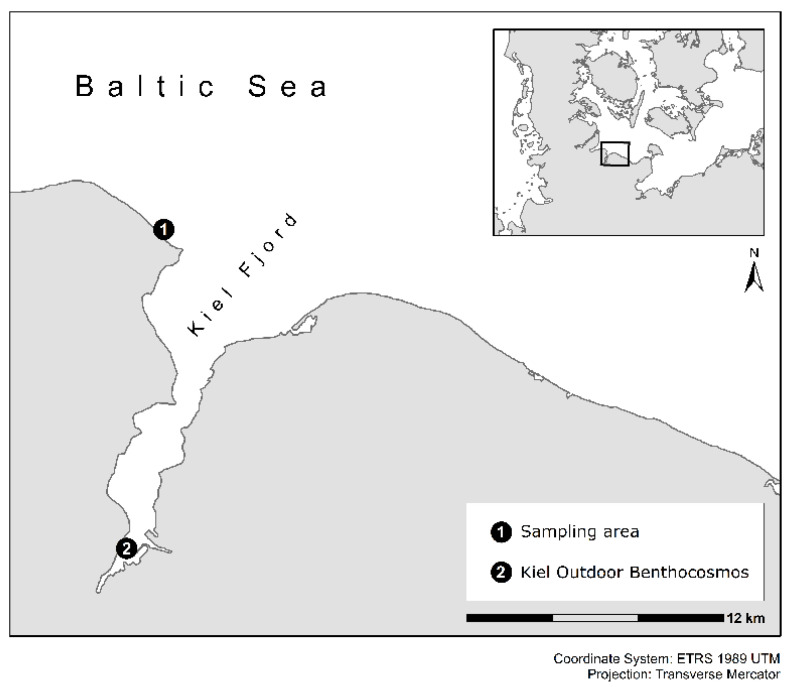
Study area in the western part of the Baltic Sea, including the location of the sampling area at Bülk as well as the position of the Kiel Outdoor Benthocosms in the Kiel Fjord, Germany.

The experiments were conducted in the Kiel Outdoor Benthocosm (KOB) infrastructure at GEOMAR in the inner Kiel Fjord (54°20′ N; 10°09′ E) (Figure 1). The detailed technical description of the KOB infrastructure, the experimental setup, and observation, can be found in Wahl et al. [62]. Briefly summarized, the KOB system is comprised of 12 tanks with a water volume of 1470 L and a flow-through of ca. 1800 L per day. The tanks are completely autonomous experimental units covered with air-tight, transparent foils. The natural weather and solar conditions affect the experimental units all year round. The experimental units were filled with nonfiltered Kiel Fjord water taken in close vicinity to the KOB infrastructure and were moved by a circulation pump within the tank to achieve water conditions comparable to the ambient conditions of the Kiel Fjord.

Temperature and pCO_2_ can be monitored and controlled in each experimental unit. Water temperature was continuously measured and automatically regulated by heat exchangers and internal heaters to an increase of 5 °C in the tanks (for technical details see Wahl et al. [62]). Kiel Fjord water temperature shows a seasonal pattern typical for temperate regions, reaching maximum temperatures (24–25 °C) during summer and minimum values (4.2 °C) in January (Table S1). Seawater pCO_2_ manipulation was realized by the computer-controlled addition of pure CO_2_ into the covered atmosphere, sustaining approximately 1100 ppm in the headspace above the experimental unit. A wave generator in each experimental unit enhanced the diffusion of CO_2_ into the water. Key environmental variables such as photosynthetically active radiation (German Weather Service), salinity (GEOMAR), dissolved inorganic nitrogen (DIN) (State Agency for Agriculture, Environment and Rural Areas Schleswig-Holstein), total alkalinity (TA), and dissolved inorganic carbon (DIC) were determined regularly (Tables S1 and S2, for more details see [62]). The analyses of TA and DIC were proceeded according to Dickson et al. [63]. Water pCO_2_ of the four different experimental treatments was calculated from the routine measures of TA, DIC, pH, salinity, and temperature with the help of the CO2SYS Excel Macro sheet [64].

The Kiel Fjord pH was high (8.5) in spring/early summer and low (7.7) in autumn/winter. The pH was measured in close vicinity to the inflow of Kiel Fjord water for the supply of the KOBs. Carbonate chemistry parameters (pH on total scale, pCO_2_, TA, and DIC) in the experimental units varied according to the four treatments and seasons (Table S2). The general mean effect of CO_2_ increase from ambient (380–450 ppm) to 1050–1100 ppm in the covered atmosphere above the experimental units decreased the water pH by 0.18 ± 0.08 pH units (Figure S1). At PANGAEA^®^ data platform (https://doi.pangaea.de/10.1594/PANGAEA.842739) all abiotic variables measured during the KOB experiments are available for each experimental unit.

### 2.2. Treatments

The individual and interactive impacts of simulated warming and acidification on antioxidative properties of *F. vesiculosus* were investigated using a fully crossed design. Two temperatures (in situ Kiel Fjord temperature vs. elevated temperature Δ + 5 °C) and CO_2_ levels (ca. 400 ppm vs. ca. 1100 ppm) in four seasonal experiments were used, resulting in four different treatments: (1) in situ Kiel Fjord temperature and pCO_2_ conditions (Ambient), (2) in situ Kiel Fjord temperature with elevated pCO_2_ (+CO_2_), (3) elevated temperature Δ + 5 °C with in situ pCO_2_ (+Temp), and (4) elevated temperature Δ + 5 °C with elevated pCO_2_ (+Temp +CO_2_). In the autonomous experimental units of the KOB facility, each treatment was replicated three times. The consecutive KOB experiments, each lasting for at least 10 weeks, were conducted in spring (4 April–19 June 2013), summer (4 July–17 September 2013), autumn (10 October–18 December 2013), and winter (16 January–1 April 2014). 

### 2.3. Fucus vesiculosus Sampling and Response Variables

*F. vesiculosus* L. individuals were sampled seasonally for each experiment (spring: 2 April 2013; summer: 2 July 2013; autumn: 8 October 2013; winter: 14 January 2014) in the atidal Kiel Fjord (Bülk), western Baltic Sea, Germany (54°27′ N; 10°11.5′ E) from a depth of 0.2–1 m (Figure 1). Here, *F. vesiculosus* specimens growing on their natural rock substrata (10–15 cm in diameter) were randomly sampled, resulting in various sizes, growth forms, and maturity levels. Adult *F. vesiculosus* individuals with a thallus length of >15 cm were collected. After collection, *F. vesiculosus* individuals were placed in water-filled buckets, transported to the KOB infrastructure, and tagged for later identification.

In each experimental unit of the KOB infrastructure, 20 individually labeled *F. vesiculosus* individuals attached to stones were set in plastic dishes (Ø = 14 cm, h = 4 cm) fixed to a grating at 40 cm water depth, and they were stepwise acclimated to the final treatment conditions by slowly raising the temperature and pCO_2_ levels over one week (for details see Graiff et al. [65]).

Three *F. vesiculosus* specimens (15–25 cm long, 91 ± 30 total apices) of apparently equal vigor were selected, and the response variables (antioxidative potential, SOD, and MDA) were calculated as the average of these different individuals. The chosen individuals were growing from one holdfast and were visually free of macroscopic epibionts. After growing for at least 10 weeks under the four different treatments in every season, 2–3 cm long vegetative apices were cut off and acclimatized for 24 h to low light conditions at the respective water temperature for later detection of *F. vesiculosus’* antioxidative potential in the laboratory. To investigate the antioxidative properties (SOD activity and lipid peroxidation) of *F. vesiculosus* individuals after growing under the four different treatments, vegetative apices were cut off, cleaned, and freeze-dried in every season. Additionally, to document the initial antioxidative status of *F. vesiculosus* growing in the Kiel Fjord, cleaned vegetative apices of twelve *F. vesiculosus* specimens were freeze-dried for further analyses.

### 2.4. Assay for Detection of F. vesiculosus’ Antioxidative Potential 

Photosynthetic performance (effective quantum yield) in actinic light of *F. vesiculosus* grown under the different treatments in the KOBs was measured as an indicator for tolerance against oxidative stress in a short-term H_2_O_2_ exposure assay. Rising concentrations (0–20 mM H_2_O_2_) were applied for 30 min in 20 mL transparent plastic vessels to investigate the tolerance width of *F. vesiculosus* to high H_2_O_2_ stress, according to the approach of Dummermuth et al. [12]. The physiological response patterns suggested that *F. vesiculosus* tolerates natural H_2_O_2_ concentrations in seawater without problems. Subsequently, the photosynthetic capacity of *F. vesiculosus* under artificial H_2_O_2_ stress was investigated by measuring the variable chlorophyll-fluorescence of photosystem II (PS II). Effective quantum yield (Δ*F/Fm’*) of *F. vesiculosus* was measured via the variable chlorophyll *a* fluorescence of PSII using a portable pulse amplitude modulated fluorometer (PAM 2000, Walz, Effeltrich, Germany) as described by Hanelt et al. [66]. Effective quantum yield values of *F. vesiculosus* apices under control conditions were characteristic for individuals that were not photosynthetically inhibited and set to 100%. All presented PAM data are expressed in relation to a paired control value. A maximum effective quantum yield value of 0.75 was found for *F. vesiculosus*, which is characteristic for unstressed and physiologically viable brown algae.

### 2.5. Lipid Peroxidation, Superoxide Dismutase (SOD), and Protein Assay

The amount of malondialdehyde (MDA) equivalents, an indicator of lipid peroxidation, was examined according to Hodges et al. [67]. Approximately 25 mg of freeze-dried and powdered *F. vesiculosus* apical biomass were homogenized in 1 mL of 0.1% of trichloroacetic acid (TCA) and centrifuged at 12,000× *g* for 10 min. The aliquot of 100 µL supernatant was blended with 900 µL of 0.5% thiobarbituric acid (TBA) in 20% TCA and incubated in a water bath at 95 °C for 30 min. The reaction was interrupted by cooling on ice for 5 min, and samples were centrifuged at 10,000× *g* for 10 min at 25 °C. Absorbance at 440, 532, and 600 nm was read in a spectrophotometer (Shimadzu UV-2401 PC, Kyoto, Japan). The amount of MDA was calculated using a molar extinction coefficient for an MDA of 0.157 × 10^6^ M^−1^ cm^−1^ and corrected for soluble sugar complexes using the formulas by Hodges et al. [67]. Results are expressed as nmol MDA g^–1^ dry mass.

An approximately 1–1.5 g dry biomass of vegetative *F. vesiculosus* apices was utilized for SOD enzyme and protein assays. Samples were extracted with 15 mL N-[2-hydroxyethyl]piperazine-N′-[2-ethanesulfonic acid] (HEPES) extraction buffer (pH 7.4). The supernatant was separated from the extract by centrifugation for 15 min at 50,000× *g* at 4 °C. The supernatant was precipitated with ammonium sulfate and centrifuged for 10 min at 50,000× *g* at 4 °C. Finally, the pellets were resuspended in 1 mL HEPES buffer (pH 7.4). SOD activity was analyzed in the extracts with the SOD Assay Kit-WST (Sigma-Aldrich, Merck KGaA, Darmstadt, Germany) according to the manufacturer’s protocol and extinction was measured at 450 nm. SOD activity in the extracts was determined and computed by using an inhibition curve with pure commercial SOD (Sigma-Aldrich, Merck KGaA, Darmstadt, Germany). Total soluble protein (TSP) was measured with the *DC* Bradford Protein Assay Kit (Bio-Rad Laboratories, Hercules, CA, USA) according to the manufacturer’s instructions. Extinction was measured at 595 nm and TSP was calculated using a calibration curve prepared with commercial bovine serum albumin. Both assays were prepared for and finally measured with a microplate spectrophotometer (SpectraMax M2e, Molecular Devices, Biberach, Germany). Finally, SOD activity was expressed as units per mg of total soluble protein (TSP). Measurements of MDA formation and SOD activity were performed on triplicate samples.

### 2.6. Statistical Analyses

Before statistical analyses, all data were verified for normality with the Kolmogorov-Smirnov test and for homogeneity with the Levene’s test and transformed, if necessary, to comply with requirements. Statistically significant differences concerning the assay for the detection of the antioxidative potential were examined by a one-way ANOVA, followed by Tukey’s honest significant difference test (Tukey-HSD) to evaluate differences to the paired control value for each treatment separately. Differences of the initial status (MDA and SOD) of *F. vesiculosus* growing in its native habitat were assessed using one-way ANOVAs for the different sampling seasons followed by *post hoc* Tukey-HSD for pairwise comparisons. Differences in the MDA concentration and SOD activity of *F. vesiculosus* over time were evaluated with repeated-measures analysis of variance (rm ANOVA), with the within-subject factor time (day) and the between-subject factors pCO_2_ and temperature used for each experiment. To assess the interactive effect of temperature and pCO_2_ on MDA concentration and SOD activity at the end of every KOB experiment, two-way ANOVAs were used with temperature and pCO_2_ as fixed factors. In addition, one-way ANOVAs were executed at the end of every KOB experiment, followed by *post hoc* Tukey-HSD for pairwise comparisons of the different treatments. The four KOB experiments cannot be statistically compared for seasonal differences because abiotic and biotic conditions were too different. SPSS Statistics 22 (IBM, Armonk, NY, USA) was used for statistical analysis.

## 3. Results

### 3.1. Antioxidative Potential 

**Figure 2:**
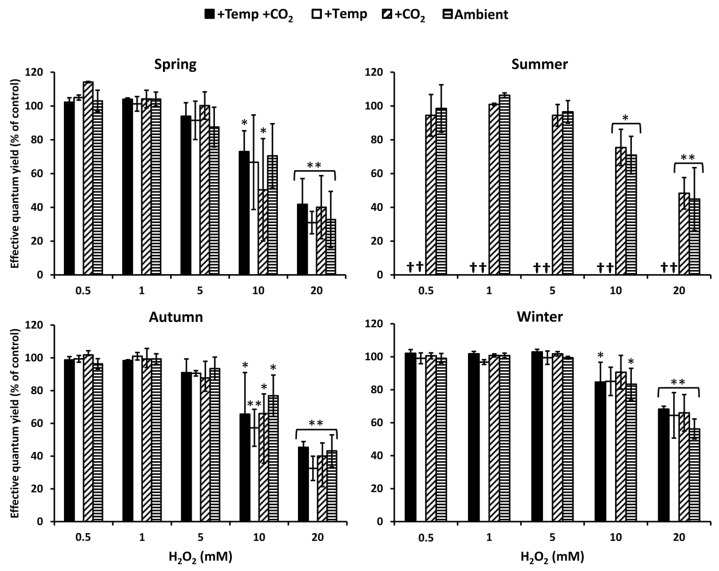
The effect of ascending H_2_O_2_ concentrations on effective quantum yield of *Fucus vesiculosus* (expressed as % of the control) grown for three months under various temperature and pCO_2_ conditions during different seasons. Seasons: spring: 4 April–19 June 2013; summer: 4 July–17 September 2013; autumn: 10 October–18 December 2013; winter: 16 January–01 April 2014. Temperature and pCO_2_ conditions: +Temp +CO_2_: elevated temperature Δ + 5°C with elevated pCO_2_; +Temp: elevated temperature Δ + 5 °C with in situ pCO_2_; +CO_2_: in situ Kiel Fjord temperature with elevated pCO_2_; Ambient: in situ Kiel Fjord temperature and pCO_2_. Values are means ± SD (standard deviation), *n* = 3. Effective quantum yield values of the controls were between 0.57 and 0.74 for all *F. vesiculosus* apices. *, **: Significant differences in comparison to the paired control value at *p* < 0.05 and *p* < 0.001, respectively (Tukey-HSD). Cross (†) marks death of *F. vesiculosus* in summer under simulated ocean warming.

Photosynthetic performance measured as effective quantum yield of *Fucus vesiculosus* under short-term H_2_O_2_ stress was regarded as an indicator for its antioxidative potential. In summer, at increased water temperatures (>26 °C), *F. vesiculosus* deceased, but during the other seasons and treatments, *F. vesiculosus* tolerated up to 5 mM H_2_O_2_, resulting in high effective quantum yields. The effective quantum yield of *F. vesiculosus* was significantly reduced, but it was still above 50% of the control in 10 mM H_2_O_2_ (Figure 2). A 50–70% reduction in effective quantum yield of *F. vesiculosus* at 20 mM was observed under all treatments in spring, summer, and autumn. The reduction of the quantum yield was stronger in spring and autumn compared to summer and winter. In winter, especially, the quantum yield was only reduced by 30–40% under all treatments in 20 mM H_2_O_2_ (Figure 2). 

### 3.2. Lipid Peroxidation

**Figure 3:**
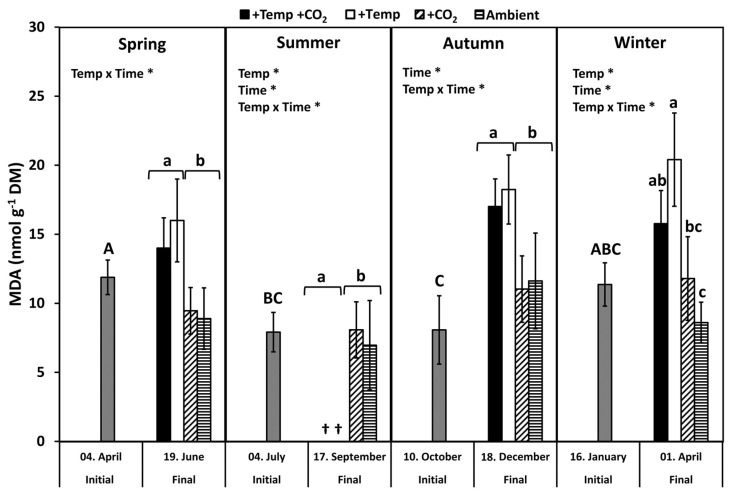
Malondialdehyde (MDA) concentration of initial *Fucus vesiculosus* growing in its native habitat (*n* = 12) and at the end of the experiments (*n* = 3), with controlled temperature and pCO_2_ conditions during different seasons. Seasons: spring: 4 April–19 June 2013; summer: 4 July–17 September 2013; autumn: 10 October–18 December 2013; winter: 16 January–1 April 2014. Temperature and pCO_2_ conditions: +Temp +CO_2_: elevated temperature Δ + 5 °C with elevated pCO_2_; +Temp: elevated temperature Δ + 5 °C with in situ pCO_2_; +CO_2_: in situ Kiel Fjord temperature with elevated pCO_2_; Ambient: in situ Kiel Fjord temperature and pCO_2_. Values are means ± SD (standard deviation). *: Significant effects of the tested factors revealed by the repeated-measure ANOVA are marked with an asterisk for each season separately. Different uppercase (comparison of initial values) and lowercase (comparison of final values between treatments) letters specify significant differences (*p* < 0.05; Tukey-HSD, data were ln-transformed in order to meet assumptions of homogeneity of variance). Cross (†) points to the death of *F. vesiculosus* in summer under simulated ocean warming.

Lipid peroxidation was examined by measuring the concentration of the biochemical marker malondialdehyde (MDA). MDA levels of all initial *F. vesiculosus* samples from its native habitat varied slightly but significantly over the course of one year (*p* < 0.05, Tukey-HSD), with higher levels in April and January compared to July and October (Figure 3). During spring, MDA content significantly increased by 20–40% among the measurement dates and under elevated temperatures (Figure 3 and Table 1). During summer, a natural heat-wave increased the Kiel Fjord water temperature dramatically [62]. Thus, the water temperature under simulated ocean warming achieved maximum levels (peak temperatures: 27–30 °C for 30 days) that surpassed the thermal tolerance of *F. vesiculosus* (>26 °C, [68]). Therefore, simulated ocean warming in combination with a summer heatwave led to a drastic dieback of the *F. vesiculosus* individuals and resulted in significant differences between the measurement dates (Figure 3 and Table 1). MDA content of *F. vesiculosus* at the end of the summer experiment was neither increased nor decreased under elevated pCO_2_ (Figure 3). Levels of MDA increased significantly in *F. vesiculosus* until the end of the autumn experiment under simulated ocean warming by 40% (Figure 3 and Table 1). In winter, the MDA content of *F. vesiculosus* almost doubled during the experiment in the increased temperature treatments (Figure 3 and Table 1). This effect of winter warming, resulting in higher MDA content in *F. vesiculosus,* was marginally alleviated at increased pCO_2_ levels; however, this effect was not significant (Figure 3). The MDA content found during the final sampling of *F. vesiculosus* in the winter experiment revealed an interactive effect of temperature and pCO_2_ (two-way ANOVA, *F* = 6.450, *df* = 1, *p* < 0.05).

### 3.3. Superoxide Dismutase

**Figure 4:**
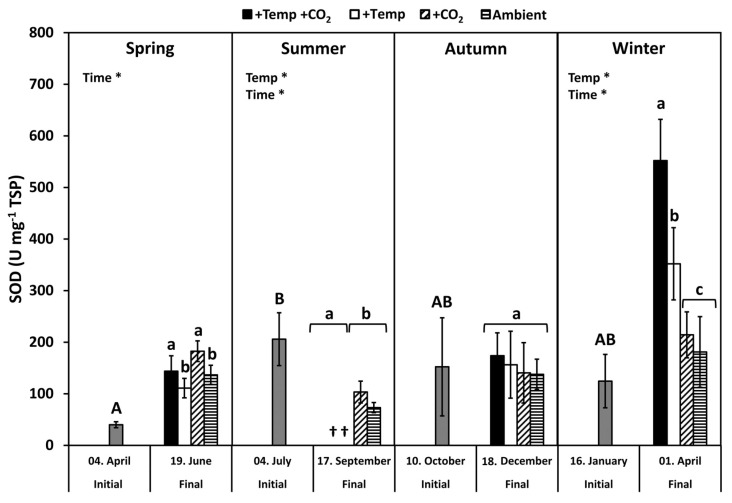
Variations of superoxide dismutase (SOD) activities of initial *Fucus vesiculosus* growing in its native habitat (*n* = 12) and at the end of the experiments (*n* = 3), with controlled temperature and pCO_2_ conditions over different seasons. Seasons: spring: 4 April–19 June 2013; summer: 4 July–17 September 2013; autumn: 10 October–18 December 2013; winter: 16 January–1 April 2014. Temperature and pCO_2_ conditions: +Temp +CO_2_: elevated temperature Δ + 5 °C with elevated pCO_2_; +Temp: elevated temperature Δ + 5 °C with in situ pCO_2_; +CO_2_: in situ Kiel Fjord temperature with elevated pCO_2_; Ambient: in situ Kiel Fjord temperature and pCO_2_. Values are means ± SD (standard deviation). *: Significant effects of the tested factors revealed by the repeated-measure ANOVA are marked with an asterisk for each season separately. Different uppercase (comparison of initial values) and lowercase (comparison of final values between treatments) letters specify significant differences (*p* < 0.05; Tukey-HSD, data were ln-transformed in order to meet assumptions of homogeneity of variance). Cross (†) points to the death of *F. vesiculosus* in summer under simulated ocean warming.

Superoxide dismutase (SOD) activity of initial *F. vesiculosus* from its native habitat was significantly higher in July than in April, but it was similar to October and January, respectively (*p* < 0.05, Tukey’s test; Figure 4). During spring, the SOD activity increased significantly among the measurement dates (Table 2). The activity of SOD was increased under elevated CO_2_ conditions by 20% at the end of the spring experiment (two-way ANOVA, *F* = 5.777, *df* = 1, *p* < 0.05). SOD activity was significantly decreased by temperature and time under summer conditions, but it was not enhanced by increased pCO_2_ under ambient temperatures (Figure 4 and Table 2). In autumn, the activity of SOD was not different between the measurement dates under all treatments. However, in winter, SOD activity increased significantly among measurement dates and under warming by 40–60% (Table 2). Maximal SOD activity of 400–500 U SOD mg^−1^ TSP was reached in winter under increased temperatures (Figure 4).

## 4. Discussion

*Fucus vesiculosus* exhibited high and relatively constant photosynthetic performance under artificial H_2_O_2_ stress. Thus, it is assumed that *F. vesiculosus* is equipped with a high antioxidative potential to tolerate strong oxidative stress, for at least short periods. High activities of SOD and a relatively low degree of lipid peroxidation (MDA concentration) found in *F. vesiculosus* over a broad range of environmental conditions were in accordance with the capability of this seaweed to endure high oxidative stress. Antioxidative properties of *F. vesiculosus* were more strongly affected by increased temperature levels than by acidification. Elevated temperature conditions significantly increased MDA concentration in *F. vesiculosus* in all seasons. However, there is a tendency that increased pCO_2_ mitigated the negative effect of elevated temperature conditions resulting in a slightly lower degree of lipid peroxidation.

### 4.1. Seasonal Response Patterns against Oxidative Stress

Seaweeds modify local water chemistry due to their metabolic photosynthesis/respiration cycles [69,70,71,72,73], and they have the potential to increase H_2_O_2_ concentrations in their habitat [24]. The thallus of the green seaweed *Ulva rigida* excretes H_2_O_2_, which can produce concentrations up to 4 µM in the experimental medium [74,75]. High concentrations of H_2_O_2_, due to excretion by seaweeds, occur under defined conditions characterized by high photosynthetic activity in areas with limited water exchange, where pH values can exceed 10 [76]. H_2_O_2_ accumulation is further supported by ultraviolet radiation (UV-B and UV-A), especially when high concentrations of DOM and oxygen occur in shallow calm waters. Thus, concentrations of H_2_O_2_ higher than 2 µM can be reached in microhabitats under specific environmental conditions [29,30]. In the present study, particularly high concentrations (≤20 mM H_2_O_2_) were applied to examine the tolerance width of *F. vesiculosus* to H_2_O_2_ stress, as the physiological response pattern of *F. vesiculosus* showed that natural H_2_O_2_ concentrations in seawater were tolerated without problems. The photosynthetic performance of *F. vesiculosus,* measured as effective quantum yield in actinic light, was used as an indicator for tolerance against oxidative stress. Studies by Collén and Pedersén [75], as well as by Dummermuth et al. [12], reported that measuring the in vivo fluorescence of photosystem II is appropriate to investigate the relative antioxidative properties of seaweeds. Short exposures to artificial H_2_O_2_ showed that the effective quantum yield of *F. vesiculosus* was high and was maintained at 0.5 and 5 mM H_2_O_2_ in all seasons. Thereafter, at 10 and 20 mM H_2_O_2_, the effective quantum yield of *F. vesiculosus* decreased by 40–70%, thus, these extremely high levels caused drastic reductions in photosynthetic rate. Dummermuth et al. [12] found a similar pattern and a high oxidative stress tolerance of Arctic *Fucus distichus* under comparable H_2_O_2_ concentrations. In contrast, other red algal and green algal seaweed species that were tested typically exhibited a pronounced H_2_O_2_ sensitivity, as was reflected in the strong inhibition of effective quantum yield at concentrations >0.5 mM [12]. At H_2_O_2_ concentrations higher than 1 mM, photosynthesis of *U. rigida* was irreversibly reduced, and this reduction was probably caused by inhibition or destruction of PS II [75]. Generally, oxidative stress-tolerant species such as *F. vesiculosus* are characterized by an effective protection system against ROS. This high tolerance of *F. vesiculosus* to H_2_O_2_ stress may be due to the accumulation of phenolic compounds such as phlorotannins. Baltic *F. vesiculosus* contains relatively high amounts of phlorotannins compared with other populations [77,78]. Phlorotannins from brown seaweeds generally possess effective antioxidant characteristics [79,80]. Thus, for brown seaweeds, phlorotannin allocation seems to act as a general stress protective feature towards various abiotic and biotic drivers [17,81]. For instance, for *Alaria esculenta*, it was found that phlorotannin allocation is a fast and cost-efficient mechanism for maintaining physiological functions under highly variable abiotic conditions [81]. Additionally, the general gene expression pattern found in response to different abiotic stressors underpin the high stress-tolerance of *F. vesiculosus* [44]. A constitutive high expression of stress-related genes (e.g., genes encoding for SOD, APX, CAT, HSPs) seem to increase the physiological tolerance of *Saccharina latissima* to abiotic stresses [42]. The increased expression of HSPs, especially, seem to play a key role in maintaining cellular homeostasis, stabilizing membranes and proteins under abiotic stress [6]. Similar mechanisms in *F. vesiculosus* may also facilitate a fast acclimation in a highly variable environment in the shallow coastal zone.

In the present study, oxidative stress was investigated directly by rises in the biomarker for lipid peroxidation (MDA) and indirectly by increasing antioxidant enzyme activities (SOD). The formation of MDA is related to intense oxidative stress that leads to cell impairment, resulting in the degradation of polyunsaturated lipids [16]. The degree of lipid peroxidation in Baltic *F. vesiculosus* peaked in April, when SOD activity (U mg^−1^ TSP) was lowest. This may indicate emerging oxidative stress in spring when the annual growth of *F. vesiculosus* commences and temperature, as well as irradiance, levels increase naturally. In general, MDA levels measured in *F. vesiculosus* in the present study were just less than half compared to *Posidonia oceanica* leaves [82]. Costa et al. [82] associated these high MDA levels with the oxidative stress of *P. oceanica* even under control conditions. High MDA levels were also found by Silva et al. [83] for other seagrass species (*Zostera marina* and *Cymodocea nodosa*), which were related to photorespiration, Mehler reaction, and cellular respiration, as well as the production of ROS. Thus, low MDA levels found in *F. vesiculosus* also point to an efficient ROS scavenging machinery and/or to a general stress protective feature such as the allocation of phlorotannins to prevent oxidative stress. Moreover, in stress-tolerant *Fucus* species (*F. spiralis*, *F. distichus*, *F. vesiculosus*) from the upper littoral zone, lipid peroxidation and reactive oxygen production connected to stressful conditions (e.g., desiccation, freezing, and high irradiances) seem to be generally low compared to more sensitive seaweed species that inhabit deeper littoral zones [10,36,84,85,86]. Low MDA levels in stressed seaweeds may also result from defensive non-enzymatic antioxidants such as different low-molecular-weight compounds (i.e., glutathione, ascorbate, carotenoids) that protect the photosynthetic apparatus from photodamage (reviewed by Mallick and Mohn [87], Rezayian et al. [88]). In the present study, these compounds were not analyzed and, thus, it is not possible to omit their contribution to the antioxidant defense system of *F. vesiculosus*. However, for fucoids, Collén and Davison [36] found that reactive oxygen scavenging enzymes are the major factor determining tolerance against oxidative stress.

Activity of the antioxidative enzyme SOD in *F. vesiculosus* was similar to that reported by other authors, such as Arctic *F. distichus* [11]. The high activities of SOD in *F. vesiculosus* are in accordance with the capability of this seaweed to resist oxidative stress. SOD activity (U mg^−1^ TSP) of *F. vesiculosus* apices showed a seasonal pattern that had the highest values in July in comparison to April, but it was similar to October and January. This pattern of SOD activity is related to the seasonally varying protein concentration, which was highest in April and lowest in July, followed by an increase from autumn to early spring (Table S3). Nitrogen in *F. vesiculosus* tracked the dissolved inorganic nitrogen (DIN) concentration in the Kiel Fjord water (Table S1) but mostly with a temporal delay. Then, nitrogen reserves, accumulated during the winter months, were fueled into growth during April to June [65,89,90,91]. However, the activities of the measured SOD in terms of U g^−1^ DM (Table S3) showed a different seasonal pattern. Using dry matter as a reference parameter, SOD activity was higher in *F. vesiculosus* apices sampled in “cold” months (i.e., mean Kiel Fjord water temperatures less than 8.5 °C; April and January) compared to “warm” months (i.e., mean Kiel Fjord water temperatures greater than 13 °C; July and October). Comparable up- and down-regulation in SOD activities resulting in seasonal patterns have been reported for different seaweed species [10,92]. Antioxidative enzymes (e.g., APX, CAT and SOD) seem to be under photoperiodic control in seaweeds [37], resulting in the highest enzyme activities in winter [84]. Temperate seaweeds seem to maintain higher enzyme activities during winter, presumably, to be equipped for rapidly rising irradiances in spring accompanied with oxidative stress. The overall pattern of seasonal alterations in antioxidative properties in *F. vesiculosus* suggests that temperature as well as light are the key factors for acclimatization and regulation of ROS metabolism [10].

### 4.2. Antioxidative Properties under Ocean Warming and Acidification

Alterations of the climate in the Baltic Sea area forecasted for the end of this century include a constant warming of surface waters by 3–6 °C, with seasonal and diurnal differences [55]. Changing temperatures impact the photosynthesis and respiration of seaweeds immediately and thereby induce the formation of ROS. Under increasing temperatures, the production of ROS is stimulated, and the antioxidant defense mechanisms will be activated to protect the seaweeds from oxidative damage [14]. Thus, metabolic pathways may acclimate to temperature changes. Elevated temperature conditions significantly increased MDA concentration in *F. vesiculosus* in spring, autumn, and winter, pointing to lipid peroxidation due to oxidative stress. During summer, under naturally increasing water temperatures and irradiances especially, a high photosynthetic activity of seaweeds also generates high concentrations of oxygen that may increase oxidative stress. However, ROS metabolism and photosynthesis are not directly related. While oxygen generates damaging ROS, it can also produce additional ATP through pseudocyclic photo-phosphorylation and the Mehler reaction [93]. Under excessive light and high temperature conditions, a strong down-regulation of several metabolic processes (e.g., photosynthesis, carbohydrate metabolism) and enhanced expression of ROS scavengers were detected for *S. latissima* by a transcriptomic analysis [45]. Heinrich et al. [45] emphasized that the harmful combination of high temperatures with high photon fluence rates caused the strongest response of *S. latissima* leading to an upregulation of programmed cell death related genes. During the summer experiment, *F. vesiculosus* individuals died as temperatures rose beyond a critical thermal threshold (>26 °C; [68]) for this species in the western Baltic Sea because a natural heat-wave intensified the elevated temperature conditions.

In summer, *F. vesiculosus* populations seem to be already threatened to decline or even completely disappear from the shallow habitats of the Baltic coast. Contrastingly, in winter, the duration of advantageous temperatures for *F. vesiculosus* growth may be prolonged due to warming [60]. Thus, Bartsch et al. [94] hypothesized that mild winter temperatures may be beneficial for the overall physiological performance of seaweeds. However, during the winter experiment, elevated temperatures increased the degree of lipid peroxidation and SOD activity significantly, indicating intensified oxidative stress for *F. vesiculosus*. The observed negative effect of experimental winter warming on overall *F. vesiculosus* performance [65,95,96] appeared contradictorily, as the experimental warming elevated temperatures from ambient 4–7 °C to 8–12 °C (December to January) and thus towards the optimal temperature range for *F. vesiculosus* growth in the western Baltic Sea (15–20 °C, [68]). These conflicting results might be explained by the energy imbalance between restricted photosynthesis at low-light winter conditions and acceleration of metabolic activity at elevated winter temperatures [97]. This energetic imbalance at the elevated winter temperature might then be enhanced by an increased production of ROS in *F. vesiculosus*, requiring biosynthesis of antioxidative enzymes indicated by increasing SOD activity. This additional energy investment in the biosynthesis of antioxidative enzymes may finally cause a reduction in photosynthetic carbon gain and growth of *F. vesiculosus* under elevated winter temperatures [65,96].

To our knowledge, there are only very few studies dealing with the effect of CO_2_-induced acidification of seawater on the antioxidative potential of micro- and macroalgae. Acidification increased the MDA content of a diatom (*Thalassiosira weissflogii*) and a brown seaweed species (*Sargassum vulgare)*, indicating that membrane lipid peroxidation was enhanced [98,99]. Additionally, the induction of SOD in *S. vulgare* under acidified conditions points to its major role in preventing ·O_2_^−^ overproduction. Kumar et al. [99] found, for *S. vulgare*, a higher energy metabolism after exposure to low pH conditions at volcanic CO_2_ vents. The study by Iñiguez et al. [100] revealed on the molecular level that CO_2_ enrichment induced only very few transcriptomic changes (e.g., induction of transcripts coding for RuBisCO *rbcL*) in the brown seaweed *Desmarestia anceps*. In spring, SOD activity in *F. vesiculosus* was significantly increased under elevated pCO_2_ conditions. However, *F. vesiculosus* did not exhibit enhanced MDA concentrations under increased pCO_2_ conditions, leading to the assumption that the formation of ROS was not induced or the antioxidant defense system of *F. vesiculosus* was quite effective in preventing lipid peroxidation. The absence of significant impacts of experimental acidification on *F. vesiculosus* antioxidative properties in this study might be explained by the fact that Kiel Fjord *F. vesiculosus* is well adapted to a high and intensely varying pCO_2_ and pH environment due to stochastic upwelling events in its natural habitat [101,102,103].

For the first time, this study presented novel details that investigated the joint impact of ocean warming and acidification on the seasonal regulation of the antioxidative potential of *F. vesiculosus*. Experimental ocean warming and acidification had no effect on the relative antioxidative potential of *F. vesiculosus* under short-term artificial H_2_O_2_ stress. During spring, autumn, and winter, higher inorganic carbon availability under elevated pCO_2_ seemed to mitigate the negative effect of elevated temperature conditions, resulting in a slightly lower degree of lipid peroxidation compared to warming alone. It was indicated that *F. vesiculosus* exhibited a more effective protection mechanism by maintaining higher SOD activity under joint warming and acidification. Thus, a higher allocation of resources to antioxidative enzymes under joint elevated temperature and pCO_2_ levels might result in a higher antioxidative activity and a lower MDA concentration in *F. vesiculosus* compared to elevated temperature levels alone.

## 5. Conclusions

This study shows that reactive oxygen metabolism plays an important role in the stress tolerance of *F. vesiculosus* under the simulated global change scenarios. Properties of the antioxidative defense system of *F. vesiculosus* against ROS were regulated seasonally. The oxidative stress was enhanced in *F. vesiculosus* under warming but seemed to be modulated by seasonally varying environmental conditions (e.g., high and low irradiances) and pCO_2_ levels. During summer, the impacts of predicted warming on *F. vesiculosus* will be most severe, as indicated by the present study and Wahl et al. [104]. The projected co-occurring increase in seawater temperatures and pCO_2_ will probably have beneficial impacts in spring, autumn, and winter on *F. vesiculosus*, as indicated by antioxidative properties (this study), photo-physiological performance [96], growth [65], and fertility [95]. Thus, at tested joint warming and acidification, *F. vesiculosus* populations may slightly benefit in distinct seasons, thereby supporting an improved start into the next period of growth. However, the occurrence and intensity of marine heatwaves during summer, reaching lethal temperatures, may restrict *F. vesiculosus* population’s persistence in the shallow coastal area of the Baltic Sea.

## Figures and Tables

**Table 1:** Repeated-measures ANOVA outcome for temperature, CO_2_, and time effects on malondialdehyde (MDA) concentration in *Fucus vesiculosus* during the different seasonal experiments (*n* = 3). Seasons: spring: 4 April–19 June 2013; summer: 4 July–17 September 2013; autumn: 10 October–18 December 2013; winter: 16 January–1 April 2014. *p*-values < 0.05 are indicated by bold type.

Source of Variation	DF	*F*-Value	*p*-Value
(a) Spring			
Temperature	1	1.078	0.33
CO_2_	1	0.188	0.68
Time	1	0.632	0.45
Temp × CO_2_	1	4.164	0.08
Temp × Time	1	5.712	**0.04**
CO_2_ × Time	1	0.536	0.49
Temp × CO_2_ × Time	1	0.264	0.62
(b) Summer			
Temperature	1	20.979	**0.002**
CO_2_	1	1.548	0.25
Time	1	22.603	**0.001**
Temp × CO_2_	1	0.002	0.96
Temp × Time	1	22.663	**0.001**
CO_2_ × Time	1	0.059	0.81
Temp × CO_2_ × Time	1	2.944	0.13
(c) Autumn			
Temperature	1	1.910	0.21
CO_2_	1	0.113	0.75
Time	1	24.796	**0.002**
Temp × CO_2_	1	0.717	0.43
Temp × Time	1	13.281	**0.008**
CO_2_ × Time	1	0.409	0.54
Temp × CO_2_ × Time	1	0.708	0.33
(d) Winter			
Temperature	1	7.368	**0.03**
CO_2_	1	0.016	0.93
Time	1	17.644	**0.003**
Temp × CO_2_	1	3.047	0.12
Temp × Time	1	18.457	**0.003**
CO_2_ × Time	1	0.381	0.55
Temp × CO_2_ × Time	1	1.989	0.19

**Table 2:** Repeated-measures ANOVA outcome for temperature, CO_2_, and time effects on superoxide dismutase (SOD) activity in *Fucus vesiculosus* during the different seasonal experiments (*n* = 3). Seasons: spring: 4 April–19 June 2013; summer: 4 July–17 September 2013; autumn: 10 October–18 December 2013; winter: 16 January–1 April 2014. *p*-values < 0.05 are indicated by bold type.

Source of variation	DF	*F*-Value	*p*-Value
(a) Spring			
Temperature	1	1.078	0.33
CO_2_	1	0.188	0.68
Time	1	5.712	**0.04**
Temp × CO_2_	1	4.464	0.08
Temp × Time	1	0.632	0.45
CO_2_ × Time	1	0.536	0.49
Temp × CO_2_ × Time	1	0.264	0.62
(b) Summer			
Temperature	1	13.536	**0.006**
CO_2_	1	0.583	0.47
Time	1	14.162	**0.006**
Temp × CO_2_	1	0.331	0.58
Temp × Time	1	3.284	0.11
CO_2_ × Time	1	1.332	0.28
Temp × CO_2_ × Time	1	0.914	0.37
(c) Autumn			
Temperature	1	2.070	0.19
CO_2_	1	0.028	0.87
Time	1	0.000	0.99
Temp × CO_2_	1	0.212	0.66
Temp × Time	1	0.090	0.77
CO_2_ × Time	1	0.019	0.89
Temp × CO_2_ × Time	1	0.014	0.91
(d) Winter			
Temperature	1	9.983	**0.01**
CO_2_	1	0.012	0.92
Time	1	12.226	**0.008**
Temp × CO_2_	1	0.044	0.84
Temp × Time	1	4.919	0.06
CO_2_ × Time	1	0.897	0.371
Temp × CO_2_ × Time	1	0.470	0.512

## Data Availability

The datasets presented in this study can be found in online repositories: PANGAEA https://doi.pangaea.de/10.1594/PANGAEA.842739.

## Supplementary Materials

The following are available online at https://www.mdpi.com/article/10.3390/biology10121330/s1, Figure S1: pH of the control and experimental units, Table S1: Abiotic parameters of the Kiel Fjord, Table S2: Seawater carbonate chemistry, and Table S3: Superoxide dismutase activity and total soluble protein content.

## References

[1] Jueterbock A., Tyberghein L., Verbruggen H., Coyer J.A., Olsen J.L., Hoarau G. (2013). Climate Change Impact on Seaweed Meadow Distribution in the North Atlantic Rocky Intertidal. Ecol. Evol..

[2] Wernberg T., Bennett S., Babcock R.C., Bettignies T.D., Cure K., Depczynski M., Dufois F., Fromont J., Fulton C.J., Hovey R.K. (2016). Climate-Driven Regime Shift of a Temperate Marine Ecosystem. Science.

[3] Smale D.A. (2020). Impacts of Ocean Warming on Kelp Forest Ecosystems. New Phytol..

[4] Dring M. (2005). Stress Resistance and Disease Resistance in Seaweeds: The Role of Reactive Oxygen Metabolism. Adv. Bot. Res..

[5] Potin P., Amsler C.D. (2008). Oxidative Burst and Related Responses in Biotic Interactions of Algae. Algal Chemical Ecology.

[6] Bischof K., Rautenberger R., Wiencke C., Bischof K. (2012). Seaweed Responses to Environmental Stress: Reactive Oxygen and Antioxidative Strategies. Seaweed Biology.

[7] Halliwell B., Gutteridge J.M.C. (2015). Free Radicals in Biology and Medicine.

[8] Asada K., Post A., Baker N.R., Bowyer J.R. (1994). Mechanisms for Scavenging Reactive Molecules Generated in Chloroplasts under Light Stress. Photoinhibition of Photosynthesis: From Molecular Mechanisms to the Field.

[9] Asada K., Foyer C., Mullineaux P.M. (1994). Production and Action of Active Oxygen Species in Photosynthetic Tissues. Causes of Photooxidative Stress and Amelioration of Defence Systems in Plants.

[10] Collén J., Davison I.R. (2001). Seasonality and Thermal Acclimation of Reactive Oxygen Metabolism in *Fucus vesiculosus* (Phaeophyceae). J. Phycol..

[11] Aguilera J., Dummermuth A., Karsten U., Schriek R., Wiencke C. (2002). Enzymatic Defences against Photooxidative Stress Induced by Ultraviolet Radiation in Arctic Marine Macroalgae. Polar Biol..

[12] Dummermuth A.L., Karsten U., Fisch K.M., König G.M., Wiencke C. (2003). Responses of Marine Macroalgae to Hydrogen-Peroxide Stress. J. Exp. Mar. Biol. Ecol..

[13] Bischof K., Rautenberger R., Brey L., Pérez-Lloréns J.L. (2006). Physiological Acclimation to Gradients of Solar Irradiance within Mats of the Filamentous Green Macroalga *Chaetomorpha linum* from Southern Spain. Mar. Ecol. Prog. Ser..

[14] Lesser M.P. (2006). Oxidative Stress in Marine Environments: Biochemistry and Physiological Ecology. Annu. Rev. Physiol..

[15] Birben E., Sahiner U.M., Sackesen C., Erzurum S., Kalayci O. (2012). Oxidative Stress and Antioxidant Defense. World Allergy Organ. J..

[16] Valenzuela A. (1991). The Biological Significance of Malondialdehyde Determination in the Assessment of Tissue Oxidative Stress. Life Sci..

[17] Cruces E., Huovinen P., Gómez I. (2012). Phlorotannin and Antioxidant Responses upon Short-Term Exposure to UV Radiation and Elevated Temperature in Three South Pacific Kelps. Photochem. Photobiol..

[18] Wei Z., Long C., Yang F., Long L., Huo Y., Ding D., Mo J. (2020). Increased Irradiance Availability Mitigates the Physiological Performance of Species of the Calcifying Green Macroalga *Halimeda* in Response to Ocean Acidification. Algal Res..

[19] Zika R.G., Moffett J.W., Petasne R.G., Cooper W.J., Saltzman E.S. (1985). Spatial and Temporal Variations of Hydrogen Peroxide in Gulf of Mexico Waters. Geochim. Cosmochim. Acta.

[20] Avery G.B., Cooper W.J., Kieber R.J., Willey J.D. (2005). Hydrogen Peroxide at the Bermuda Atlantic Time Series Station: Temporal Variability of Seawater Hydrogen Peroxide. Mar. Chem..

[21] Rusak S.A., Peake B.M., Richard L.E., Nodder S.D., Cooper W.J. (2011). Distributions of Hydrogen Peroxide and Superoxide in Seawater East of New Zealand. Mar. Chem..

[22] Szymczak R., Waite T. (1988). Generation and Decay of Hydrogen Peroxide in Estuarine Waters. Mar. Freshw. Res..

[23] Yuan J., Shiller A.M. (2001). The Distribution of Hydrogen Peroxide in the Southern and Central Atlantic Ocean. Deep-Sea Res. Part II Top. Stud. Oceanogr..

[24] Twigg I.M., Baltar F., Hall J.R., Hepburn C.D. (2020). Revealing Hydrogen Peroxide as an External Stressor in Macrophyte-Dominated Coastal Ecosystems. Oecologia.

[25] Cooper W.J., Zika R.G., Petasne R.G., Plane J.M.C. (1988). Photochemical Formation of H_2_O_2_ in Natural Waters Exposed to Sunlight. Environ. Sci. Technol..

[26] Mopper K., Zhou X. (1990). Hydroxyl Radical Photoproduction in the Sea and Its Potential Impact on Marine Processes. Science.

[27] O’Sullivan D.W., Neale P.J., Coffin R.B., Boyd T.J., Osburn C.L. (2005). Photochemical Production of Hydrogen Peroxide and Methylhydroperoxide in Coastal Waters. Mar. Chem..

[28] Garg S., Rose A.L., Waite T.D. (2011). Photochemical Production of Superoxide and Hydrogen Peroxide from Natural Organic Matter. Geochim. Cosmochim. Acta.

[29] Abele-Oeschger D., Tüg H., Röttgers R. (1997). Dynamics of UV-Driven Hydrogen Peroxide Formation on an Intertidal Sandflat. Limnol. Oceanogr..

[30] Abele D., Ferreyra G.A., Schloss I. (1999). H_2_O_2_ Accumulation from Photochemical Production and Atmospheric Wet Deposition in Antarctic Coastal and Off-Shore Waters of Potter Cove, King George Island, South Shetland Islands. Antarct. Sci..

[31] Winterbourn C.C., Abelson J.N., Simon M.I. (2013). The Biological Chemistry of Hydrogen Peroxide. Methods in Enzymology.

[32] Badger M. (2003). The Roles of Carbonic Anhydrases in Photosynthetic CO_2_ Concentrating Mechanisms. Photosynth. Res..

[33] Parry M.A.J., Keys A.J., Madgwick P.J., Carmo-Silva A.E., Andralojc P.J. (2008). Rubisco Regulation: A Role for Inhibitors. J. Exp. Bot..

[34] Tcherkez G. (2016). The Mechanism of Rubisco-Catalysed Oxygenation. Plant. Cell Environ..

[35] Foyer C.H., Lopez-Delgado H., Dat J.F., Scott I.M. (1997). Hydrogen Peroxide- and Glutathione-Associated Mechanisms of Acclimatory Stress Tolerance and Signalling. Physiol. Plant..

[36] Collén J., Davison I.R. (1999). Reactive Oxygen Production and Damage in Intertidal *Fucus* Spp. (Phaeophyceae). J. Phycol..

[37] Carvalho A.M., Neto A.M.P., Tonon A.P., Pinto E., Cardozo K.H.M., Brigagão M.R.P.L., Barros M.P., Torres M.A., Magalhães P., Cg S. (2004). Circadian Protection against Oxidative Stress in Marine Algae. Hypnos.

[38] Takahashi M.-A., Asada K. (1983). Superoxide Anion Permeability of Phospholipid Membranes and Chloroplast Thylakoids. Arch. Biochem. Biophys..

[39] Ross C., Alstyne K.L.V. (2007). Intraspecific Variation in Stress-Induced Hydrogen Peroxide Scavenging by the Ulvoid Macroalga *Ulva lactuca*. J. Phycol..

[40] Dittami S.M., Scornet D., Petit J.-L., Ségurens B., Da Silva C., Corre E., Dondrup M., Glatting K.-H., König R., Sterck L. (2009). Global Expression Analysis of the Brown Alga *Ectocarpus siliculosus* (Phaeophyceae) Reveals Large-Scale Reprogramming of the Transcriptome in Response to Abiotic Stress. Genome Biol..

[41] Liu F., Wang W., Sun X., Liang Z., Wang F. (2014). RNA-Seq Revealed Complex Response to Heat Stress on Transcriptomic Level in *Saccharina japonica* (Laminariales, Phaeophyta). J. Appl. Phycol..

[42] Li H., Monteiro C., Heinrich S., Bartsch I., Valentin K., Harms L., Glöckner G., Corre E., Bischof K. (2020). Responses of the Kelp *Saccharina latissima* (Phaeophyceae) to the Warming Arctic: From Physiology to Transcriptomics. Physiol. Plant..

[43] Collén J., Guisle-Marsollier I., Léger J.J., Boyen C. (2007). Response of the Transcriptome of the Intertidal Red Seaweed *Chondrus crispus* to Controlled and Natural Stresses. New Phytol..

[44] Pearson G.A., Hoarau G., Lago-Leston A., Coyer J.A., Kube M., Reinhardt R., Henckel K., Serrão E.A., Corre E., Olsen J.L. (2010). An Expressed Sequence Tag Analysis of the Intertidal Brown Seaweeds *Fucus serratus* (L.) and *F. vesiculosus* (L.) (Heterokontophyta, Phaeophyceae) in Response to Abiotic Stressors. Mar. Biotechnol..

[45] Heinrich S., Valentin K., Frickenhaus S., John U., Wiencke C. (2012). Transcriptomic Analysis of Acclimation to Temperature and Light Stress in *Saccharina latissima* (Phaeophyceae). PLoS ONE.

[46] Kautsky H., Kautsky L., Kautsky N., Kautsky U., Lindblad C., Wallentinus I., Snoeijs P. (1992). Studies on the *Fucus vesiculosus* Community in the Baltic Sea. Phycological Studies of Nordic Coastal Waters.

[47] Rönnbäck P., Kautsky N., Pihl L., Troell M., Söderqvist T., Wennhage H. (2007). Ecosystem Goods and Services from Swedish Coastal Habitats: Identification, Valuation, and Implications of Ecosystem Shifts. Ambio.

[48] Wahl M., Shahnaz L., Dobretsov S., Saha M., Symanowski F., David K., Lachnit T., Vasel M., Weinberger F. (2010). Ecology of Antifouling Resistance in the Bladder Wrack *Fucus vesiculosus*: Patterns of Microfouling and Antimicrobial Protection. Mar. Ecol. Prog. Ser..

[49] Wahl M., Jormalainen V., Eriksson B.K., Coyer J.A., Molis M., Schubert H., Dethier M., Karez R., Kruse I., Lenz M., Lesser M. (2011). Stress Ecology in *Fucus*: Abiotic, Biotic and Genetic Interactions. Advances in Marine Biology.

[50] Kautsky H., van der Maarel E. (1990). Multivariate Approaches to the Variation in Phytobenthic Communities and Environmental Vectors in the Baltic Sea. Mar. Ecol. Prog. Ser..

[51] Jormalainen V., Wikström S.A., Honkanen T. (2008). Fouling Mediates Grazing: Intertwining of Resistances to Multiple Enemies in the Brown Alga *Fucus vesiculosus*. Oecologia.

[52] Werner F.J., Graiff A., Matthiessen B. (2016). Temperature Effects on Seaweed-Sustaining Top-down Control Vary with Season. Oecologia.

[53] Torn K., Krause-Jensen D., Martin G. (2006). Present and Past Depth Distribution of Bladderwrack (*Fucus vesiculosus*) in the Baltic Sea. Aquat. Bot..

[54] Takolander A., Cabeza M., Leskinen E. (2017). Climate Change Can Cause Complex Responses in Baltic Sea Macroalgae: A Systematic Review. J. Sea Res..

[55] Elken J., Lehmann A., Myrberg K., The BACC II Author Team (2015). Recent Change-Marine Circulation and Stratification. Second Assessment of Climate Change for the Baltic Sea Basin.

[56] Hobday A.J., Alexander L.V., Perkins S.E., Smale D.A., Straub S.C., Oliver E.C.J., Benthuysen J.A., Burrows M.T., Donat M.G., Feng M. (2016). A Hierarchical Approach to Defining Marine Heatwaves. Prog. Oceanogr..

[57] HELCOM (2013). Climate Change in the Baltic Sea Area: HELCOM Thematic Assessment in 2013. Baltic Sea Environment Proceedings.

[58] Müller J.D., Schneider B., Rehder G. (2016). Long-Term Alkalinity Trends in the Baltic Sea and Their Implications for CO_2_-Induced Acidification. Limnol. Oceanogr..

[59] Omstedt A., Edman M.K., Claremar B., Frodin P., Gustafsson E., Humborg C., Hägg H., Mörth M., Rutgersson A., Schurgers G. (2012). Future Changes in the Baltic Sea Acid-Base (PH) and Oxygen Balances. Tellus.

[60] Bolle H.-J., Menenti M., Rasool S.I., BACC II Author Team (2015). Second Assessment of Climate Change for the Baltic Sea Basin.

[61] Schneider B., Eilola K., Lukkari K., Müller-Karulis B., Neumann T., The BACC II Author Team (2015). Environmental Impacts—Marine Biogeochemistry. Second Assessment of Climate Change for the Baltic Sea Basin.

[62] Wahl M., Buchholz B., Winde V., Golomb D., Guy-Haim T., Müller J., Rilov G., Scotti M., Böttcher M.E. (2015). A Mesocosm Concept for the Simulation of Near-Natural Shallow Underwater Climates: The Kiel Outdoor Benthocosms (KOB). Limnol. Oceanogr. Methods.

[63] Dickson A.G., Sabine C.L., Christian J.R. (2007). Guide to Best Practices for Ocean. CO_2_ Measurements. PICES Special Publication 3.

[64] Pierrot D., Lewis E., Wallace D.W.R. (2006). MS Excel Program. Developed for CO_2_ System Calculations.

[65] Graiff A., Bartsch I., Ruth W., Wahl M., Karsten U. (2015). Season Exerts Differential Effects of Ocean Acidification and Warming on Growth and Carbon Metabolism of the Seaweed *Fucus vesiculosus* in the Western Baltic Sea. Front. Mar. Sci..

[66] Hanelt D. (1998). Capability of Dynamic Photoinhibition in Arctic Macroalgae Is Related to Their Depth Distribution. Mar. Biol..

[67] Hodges D.M., DeLong J.M., Forney C.F., Prange R.K. (1999). Improving the Thiobarbituric Acid-Reactive-Substances Assay for Estimating Lipid Peroxidation in Plant Tissues Containing Anthocyanin and Other Interfering Compounds. Planta.

[68] Graiff A., Liesner D., Karsten U., Bartsch I. (2015). Temperature Tolerance of Western Baltic Sea *Fucus vesiculosus*—Growth, Photosynthesis and Survival. J. Exp. Mar. Biol. Ecol..

[69] Gaylord B., Rosman J.H., Reed D.C., Koseff J.R., Fram J., MacIntyre S., Arkema K., McDonald C., Brzezinski M.A., Largier J.L. (2007). Spatial Patterns of Flow and Their Modification within and around a Giant Kelp Forest. Limnol. Oceanogr..

[70] Cornwall C.E., Hepburn C.D., Pilditch C.A., Hurd C.L. (2013). Concentration Boundary Layers around Complex Assemblages of Macroalgae: Implications for the Effects of Ocean Acidification on Understory Coralline Algae. Limnol. Oceanogr..

[71] Hurd C.L. (2015). Slow-Flow Habitats as Refugia for Coastal Calcifiers from Ocean Acidification. J. Phycol..

[72] Wahl M., Schneider Covachã S., Saderne V., Hiebenthal C., Müller J.D., Pansch C., Sawall Y. (2018). Macroalgae May Mitigate Ocean Acidification Effects on Mussel Calcification by Increasing pH and its Fluctuations. Limnol. Oceanogr..

[73] Murie K.A., Bourdeau P.E. (2020). Fragmented Kelp Forest Canopies Retain Their Ability to Alter Local Seawater Chemistry. Sci. Rep..

[74] Collén J., Del Rio M.J., García-Reina G., Pedersén M. (1995). Photosynthetic Production of Hydrogen Peroxide by *Ulva rigida* C. Ag. (Chlorophyta). Planta.

[75] Collén J., Pedersén M. (1996). Production, Scavenging and Toxicity of Hydrogen Peroxide in the Green Seaweed *Ulva rigida*. Eur. J. Phycol..

[76] Truchot J.-P., Duhamel-Jouve A. (1980). Oxygen and Carbon Dioxide in the Marine Intertidal Environment: Diurnal and Tidal Changes in Rockpools. Respirin Physiol..

[77] Jormalainen V., Honkanen T., Amsler C.D. (2008). Macroalgal Chemical Defenses and Their Roles in Structuring Temperate Marine Communities. Algal Chemical Ecology.

[78] Kubanek J., Lester S., Fenical W., Hay M. (2004). Ambiguous Role of Phlorotannins as Chemical Defenses in the Brown Alga *Fucus vesiculosus*. Mar. Ecol. Prog. Ser..

[79] Shin T., Ahn M., Hyun J.W., Kim S.H., Moon C. (2014). Antioxidant Marine Algae Phlorotannins and Radioprotection: A Review of Experimental Evidence. Acta Histochem..

[80] Ahn G.-N., Kim K.-N., Cha S.-H., Song C.-B., Lee J., Heo M.-S., Yeo I.-K., Lee N.-H., Jee Y.-H., Kim J.-S. (2007). Antioxidant Activities of Phlorotannins Purified from *Ecklonia cava* on Free Radical Scavenging Using ESR and H_2_O_2_-Mediated DNA Damage. Eur. Food Res. Technol..

[81] Springer K., Lütz C., Lütz-Meindl U., Wendt A., Bischof K. (2017). Hyposaline Conditions Affect UV Susceptibility in the Arctic Kelp *Alaria esculenta* (Phaeophyceae). Phycologia.

[82] Costa M.M., Barrote I., Silva J., Olivé I., Alexandre A., Albano S., Santos R.O.P. (2015). Epiphytes Modulate Posidonia Oceanica Photosynthetic Production, Energetic Balance, Antioxidant Mechanisms and Oxidative Damage. Front. Mar. Sci..

[83] Silva J., Barrote I., Costa M.M., Albano S., Santos R. (2013). Physiological Responses of *Zostera marina* and *Cymodocea nodosa* to Light-Limitation Stress. PLoS ONE.

[84] Dummermuth A. (2003). Antioxidative Properties of Marine Macroalgae from the Arctic. Ph.D. Thesis.

[85] Collén J., Davison I.R. (1999). Stress Tolerance and Reactive Oxygen Metabolism in the Intertidal Red Seaweeds *Mastocarpus stellatus* and *Chondrus crispus*. Plant. Cell Environ..

[86] Collén J., Davison I.R. (1999). Reactive Oxygen Metabolism in Intertidal *Fucus* spp. (Phaeophyceae). J. Phycol..

[87] Mallick N., Mohn F.H. (2000). Reactive Oxygen Species: Response of Algal Cells. J. Plant. Physiol..

[88] Rezayian M., Niknam V., Ebrahimzadeh H. (2019). Oxidative Damage and Antioxidative System in Algae. Toxicol. Rep..

[89] Carlson L. (1991). Seasonal Variation in Growth, Reproduction and Nitrogen Content of *Fucus vesiculosus* L. in the Öresund, Southern Sweden. Bot. Mar..

[90] Pedersen M.F., Borum J. (1996). Nutrient Control of Algal Growth in Estuarine Waters. Nutrient Limitation and the Importance of Nitrogen Requirements and Nitrogen Storage among Phytoplankton and Species of Macroalgae. Mar. Ecol. Prog. Ser..

[91] Lehvo A., Bäck S., Kiirikki M. (2001). Growth of *Fucus vesiculosus* L. (Phaeophyta) in the Northern Baltic Proper: Energy and Nitrogen Storage in Seasonal Environment. Bot. Mar..

[92] Lohrmann N.L., Logan B.A., Johnson A.S. (2004). Seasonal Acclimatization of Antioxidants and Photosynthesis in *Chondrus crispus* and *Mastocarpus stellatus*, Two Co-Occurring Red Algae with Differing Stress Tolerances. Biol. Bull..

[93] Asada K., Takahashi M.-A., Kyle D.J., Osmond C.B., Arntzen C.J. (1987). Production and Scavenging of Active Oxygen in Photosynthesis. Photoinhibition.

[94] Bartsch I., Wiencke C., Laepple T., Wiencke C., Bischof K. (2012). Global Seaweed Biogeography under a Changing Climate: The Prospected Effects of Temperature. Seaweed Biology.

[95] Graiff A., Dankworth M., Wahl M., Karsten U., Bartsch I. (2017). Seasonal Variations of *Fucus vesiculosus* Fertility under Ocean Acidification and Warming in the Western Baltic Sea. Bot. Mar..

[96] Graiff A., Bartsch I., Glaser K., Karsten U. (2021). Seasonal Photophysiological Performance of Adult Western Baltic *Fucus vesiculosus* (Phaeophyceae) under Ocean Warming and Acidification. Front. Mar. Sci..

[97] Rohde S., Hiebenthal C., Wahl M., Karez R., Bischof K. (2008). Decreased Depth Distribution of *Fucus vesiculosus* (Phaeophyceae) in the Western Baltic: Effects of Light Deficiency and Epibionts on Growth and Photosynthesis. Eur. J. Phycol..

[98] Liu F.J., Li S.X., Huang B.Q., Zheng F.Y., Huang X.G. (2016). Effect of Excessive CO_2_ on Physiological Functions in Coastal Diatom. Sci. Rep..

[99] Kumar A., AbdElgawad H., Castellano I., Lorenti M., Delledonne M., Beemster G.T.S., Asard H., Buia M.C., Palumbo A. (2017). Physiological and Biochemical Analyses Shed Light on the Response of *Sargassum vulgare* to Ocean Acidification at Different Time. Front. Plant. Sci..

[100] Iñiguez C., Heinrich S., Harms L., Gordillo F.J.L. (2017). Increased Temperature and CO_2_ Alleviate Photoinhibition in *Desmarestia anceps*: From Transcriptomics to Carbon Utilization. J. Exp. Bot..

[101] Thomsen J., Gutowska M.A., Saphörster J., Heinemann A., Trübenbach K., Fietzke J., Hiebenthal C., Eisenhauer A., Körtzinger A., Wahl M. (2010). Calcifying Invertebrates Succeed in a Naturally CO_2_-Rich Coastal Habitat but Are Threatened by High Levels of Future Acidification. Biogeosciences.

[102] Melzner F., Thomsen J., Koeve W., Oschlies A., Gutowska M.A., Bange H.W., Hansen H.P., Körtzinger A. (2013). Future Ocean Acidification Will Be Amplified by Hypoxia in Coastal Habitats. Mar. Biol..

[103] Saderne V. (2012). The Ecological Effect of CO2 on the Brown Algae *Fucus serratus* and Its Epibionts: From the Habitat to the Organismic Scale. Ph.D. Thesis.

[104] Wahl M., Werner F.J., Buchholz B., Raddatz S., Graiff A., Matthiessen B., Karsten U., Hiebenthal C., Hamer J., Ito M. (2019). Season Affects Strength and Direction of the Interactive Impacts of Ocean Warming and Biotic Stress in a Coastal Seaweed Ecosystem. Limnol. Oceanogr..

