# Supplementary material for: Antioxidative Properties of Baltic Sea Keystone Macroalgae (Fucus vesiculosus, Phaeophyceae) under Ocean Warming and Acidification in a Seasonally Varying Environment
Source: Biology (Basel). 2021 Dec 15;10(12):1330. doi: 10.3390/biology10121330 (PMC8698884; doi:10.3390/biology10121330)
Supplement: Supplementary file 1 [file biology-10-01330-s001.zip › biology-1492692-supplementary.pdf]

Supplement

Table S1 Photosynthetically active radiation (PAR) at the surface, dissolved inorganic nitrogen (DIN) in the Kiel Fjord as well as mean, minimum, and maximum Benthocosm temperatures in each month (*n* = 3, mean ± SD).

		PAR (µmol m^{-2} s^{-1})	DIN (mg l^{-1})	temperature in the Kiel Fjord (°C)			temperature in the Kiel Fjord +5°C		
		mean	mean	mean±SD	min	max	mean±SD	min	max
Spring 2013	April	327	0.1045	8.2±1.4	4.3	11.7	11.3±1.1	9.0	18.4
	May	415	0.0774	11.3±2.1	6.1	17.4	15.4±2.8	10.6	21.7
	June	641	0.0102	16.1±1.3	11.7	19.4	21.1±1.3	15.5	23.7
Summer 2013	July	616	0.0020	20.4±1.8	14.8	24.1	25.0±2.5	16.6	29.0
	August	516	0.0312	19.5±1.2	14.7	24.8	24.5±1.1	19.5	30.0
	September	315	0.0251	17.4±1.0	13.3	19.6	22.4±1.0	18.3	24.7
Autumn 2013	October	139	0.0227	13.1±0.5	11.6	14.6	17.7±0.9	12.7	19.0
	November	78	0.1941	10.3±1.6	7.2	13.1	15.2±1.6	12.1	18.1
	December	36	0.3294	6.9±0.5	5.5	9.5	11.8±0.5	9.7	13.3
Winter 2014	January	68	0.2241	4.2±1.4	1.7	7	7.8±1.3	5.4	10.6
	February	195	0.4984	4.3±0.7	2.3	6.3	8.4±0.9	6.9	11.0
	March	263	0.1923	6.7±1.1	4.4	9.1	11.5±1.2	7.8	14.4

Table S2 Summary of mean monthly seawater carbonate chemistry. pCO_2 (*n* = 3, ± SD) was calculated from total alkalinity (TA, *n* = 3), dissolved organic carbon (DIC, *n* = 3) and pH on total scale (*n* = 3) measurements of seawater corresponding to each treatment (Wahl et al. 2015 [62], M. Böttcher and V. Winde pers. comm.).

			Spring 2013			Summer 2013			Autumn 2013			Winter 2014		
			April	May	June	July	August	September	October	November	December	January	February	March
Ambient	pH		8.60±0.11	8.14±0.40	8.13±0.39	8.07±0.19	7.83±0.11	7.78±0.12	7.84±0.09	7.84±0.08	7.74±0.05	7.83±0.07	7.89±0.08	7.95±0.14
	pCO_2	ppm	130±57	516±413	606±390	489±228	772±254	886±284	723±159	719±163	877±71	718±113	636±85	587±219
	TA	µmol kg^{-1}	2066±82	2007±69	2059±91	1971±38	1906±103	1901±38	1968±33	2058±57	2077±14	2223±44	2173±12	2142±55
	DIC	µmol kg^{-1}	1731	1818	1917	1927	1849	1890	1950	2054	2033	2208	2070	2201
+CO_2	pH		8.33±0.16	7.93 ±0.39	7.82±0.43	7.87±0.17	7.70±0.10	7.62±0.13	7.74±0.09	7.72±0.09	7.60±0.05	7.69±0.07	7.66±0.04	7.69±0.07
	pCO_2	ppm	297±192	854±663	1244±815	786±320	1070±333	1313±397	924±198	927±212	1217±142	1066±57	1087±94	1061±210
	TA	µmol kg^{-1}	2069±87	2008±70	2069±87	1972±45	1916±112	1910±39	1979±39	2052±56	2086±17	2197±53	2174±10	2143±54
	DIC	µmol kg^{-1}	1991	1883	2013	1969	1883	1921	1970	2085	2071	2248	2178	2221
+Temp	pH		8.39±0.12	7.90±0.38	8.00±0.18	7.91 ±0.26	7.61±0.13	7.60±0.09	7.73±0.07	7.76±0.08	7.71±0.03	7.79±0.07	7.78±0.04	7.70±0.10
	pCO_2	ppm	220±121	960±837	656±244	828±538	1414±531	1373±343	952±166	878±160	953±81	808±142	826±83	1074±313
	TA	µmol kg^{-1}	2067±85	2005±71	2051±93	1975±36	1921±100	1914±38	1979±31	2053±44	2081±18	2205±48	2166±9	2106±69
	DIC	µmol kg^{-1}	1929	1905	2069	1926	1876	1934	1956	2055	2034	2208	2122	2160
+Temp +CO_2	pH		8.14±0.19	7.71±0.38	7.73±0.25	7.78±0.25	7.50±0.13	7.50±0.10	7.68±0.11	7.68±0.08	7.56±0.04	7.68±0.09	7.60±0.04	7.53±0.07
	pCO_2	ppm	503±402	1200±947	1357±640	1385±809	1725±556	1490±885	1118±290	1092±202	1384±107	1071±215	1291±147	1585±263
	TA	µmol kg^{-1}	2095±89	2022±79	2059±85	1964±48	1905±108	1912±42	1976±44	2065±54	2077±8	2217±40	2168±13	2111±69
	DIC	µmol kg^{-1}	2050	1953	2119	1907	1959	1970	1978	2083	2073	2244	2164	2197

Table S3 Initial superoxide dismutase (SOD) activity and total soluble protein (TSP) content of *Fucus vesiculosus* apices in its native habitat (initial, n = 12) and after growing for 3 months in different seasons, temperature, and pCO_2 conditions in the Benthocosms (n = 3). Seasons: spring: 04.04-19.06.2013; summer: 04.07-17.09.2013; autumn: 10.10-18.12.2013; winter: 16.01-01.04.2014. Temperature and pCO_2 conditions: +Temp +CO_2: elevated temperature Δ+5°C with elevated pCO_2, +Temp: elevated temperature Δ+5°C with *in situ* pCO_2, +CO_2: *in situ* Kiel Fjord temperature with elevated pCO_2, Ambient: *in situ* Kiel Fjord temperature and pCO_2. Values are means ± SD (standard deviation). Cross (†) indicates dieback of *F. vesiculosus* in the summer experiment under warming.

			SOD ($U\ g^{-1}$ DM)	TSP ($mg\ g^{-1}$ DM)
Spring	April	Initial	67.0±9.5	171.2±33.0
	June	+Temp +CO_2	78.5±9.4	56.1±13.2
		+Temp	80.4±2.8	73.5±10.1
		+CO_2	94.3±13.0	53.0±10.8
		Ambient	87.7±10.3	65.1±13.4
Summer	July	Initial	22.6±16.2	25.3±27.6
	September	+Temp +CO_2	†	†
		+Temp	†	†
		+CO_2	20.6±2.0	20.6±5.2
		Ambient	15.7±1.7	21.5±0.7
Autumn	October	Initial	38.6±12.3	68.5±35.6
	December	+Temp +CO_2	90.0±16.3	53.8±14.3
		+Temp	94.8±16.0	65.0±16.1
		+CO_2	71.8±19.8	60.7±34.7
		Ambient	80.8±14.9	59.3±8.2
Winter	January	Initial	84.5±26.9	74.3±30.4
	April	+Temp +CO_2	168.4±57.2	31.4±11.0
		+Temp	129.8±15.7	46.3±34.8
		+CO_2	149.5±30.6	71.3±16.1
		Ambient	127.9±11.9	76.8±24.4

Figure S1 (a) pH in the control (blue) and +CO_2 (red) experimental units of a tank with ambient temperature and in Kiel Fjord (*in situ*, black). (b) pH in the +Temp (blue) and +Temp +CO_2 (red) experimental units of a tank with increased temperature and in Kiel Fjord (*in situ*, black). Note that Kiel Fjord pH measurements prior to 30 May were taken at a shallower depth than the inlet of the flow-through. After that date pH in Kiel Fjord was measured close to the inlet (modified according to Wahl et al. 2015 [62]).

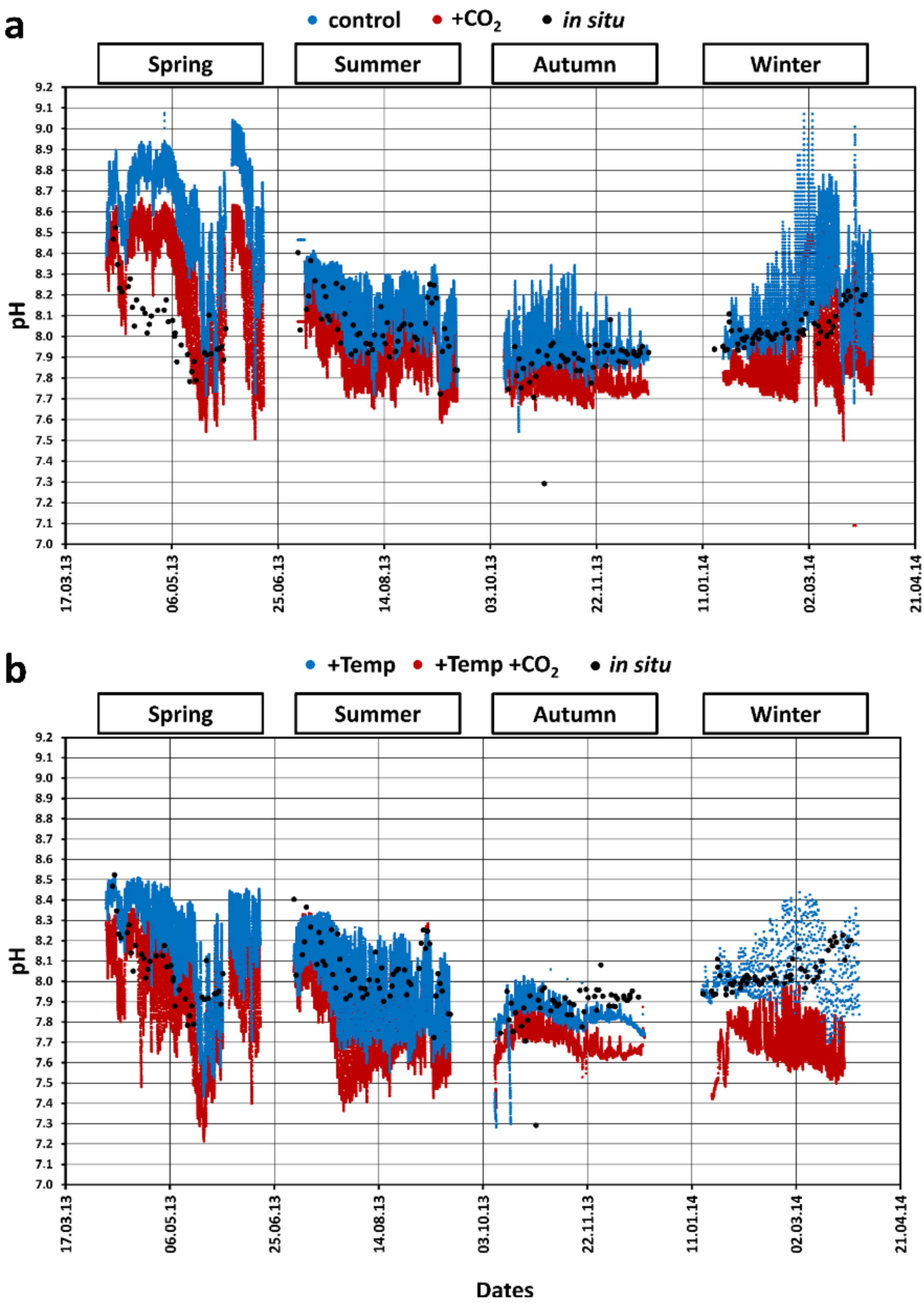
a
control
$+CO_2$
in situ
Spring
Summer
Autumn
Winter
pH
9.2
9.1
9.0
8.9
8.8
8.7
8.6
8.5
8.4
8.3
8.2
8.1
8.0
7.9
7.8
7.7
7.6
7.5
7.4
7.3
7.2
7.1
7.0
17.03.13
06.05.13
25.06.13
14.08.13
03.10.13
22.11.13
11.01.14
02.03.14
21.04.14
b
+Temp
$+Temp +CO_2$
in situ
Spring
Summer
Autumn
Winter
pH
Dates

Figure S1
